# SPE/TLC/Densitometric Quantification of Selected Synthetic Food Dyes in Liquid Foodstuffs and Pharmaceutical Preparations

**DOI:** 10.1155/2017/9528472

**Published:** 2017-07-17

**Authors:** Anna W. Sobańska, Jarosław Pyzowski, Elżbieta Brzezińska

**Affiliations:** Department of Analytical Chemistry, Medical University of Lodz, Ul. Muszynskiego 1, 90-151 Lodz, Poland

## Abstract

Selected synthetic food dyes (tartrazine, Ponceau 4R, Brilliant Blue, orange yellow, and azorubine) were isolated from liquid preparations (mouthwashes and beverages) by Solid Phase Extraction on aminopropyl-bonded silica with diluted aqueous sodium hydroxide as an eluent. The extraction step was followed by thin layer chromatography on silica gel 60 with chloroform-isopropanol-25% aq. ammonia 1 : 3 : 1 (v/v/v) as mobile phase and the densitometric quantification of dyes was achieved using quadratic calibration plots (*R*^2^ > 0.997; LOQ = 0.04–0.09 *μ*gspot^−1^). The overall recoveries for all studied dyes were at the average level of over 90% and the repeatability of the proposed procedure (CV ≤ 4.1%) was sufficient to recommend it for the routine quantification of the aforementioned dyes in liquid matrices.

## 1. Introduction

Synthetic food dyes are still common food additives despite the growing awareness of their negative influence on the human organism. The particularly harmful food colorants are azo dyes that exhibit carcinogenic and potentially genotoxic activity [1]. The list of dyes permitted in the European Union contains over 30 substances of which 12 are synthetic colorants [2].

Legal requirements and limitations regarding the application of food dyes have led to the development of several analytical techniques that enable the detection and quantification of these food additives. Liquid chromatography, including thin layer chromatography (TLC) in normal (NP) and reversed (RP) phase mode, has been successfully used to quantify food dyes in different matrices. Selected references on TLC methods proposed to separate and quantify permitted and illegal synthetic food dyes are listed in Table 1.

The actual determination of colorants in different food matrices was often preceded by the sample pretreatment stage including Solid Phase Extraction (SPE) on sorbents such as cellulose (cotton wool) [3], octadecyl-bonded silica (RP-18) [4–7], polyamide powder [8], alumina [9], polyurethane foam [10], or amino-modified silica (NH_2_) [5]. Quantification of dyes, separated by TLC, was achieved by sorbent scraping, extraction, and spectrophotometric analysis of extracts [11, 12], mass spectroscopy [13], densitometry [4, 5, 12, 14], or software processing of images scanned with flatbed scanners [15–18].

The purpose of this study was to develop a new, easy, and rapid methodology that could be used to quantify the most common synthetic dyestuffs in liquid matrices such as beverages or mouthwashes using the cheapest possible chromatographic plates and a simple sample clean-up step.

## 2. Experimental

### 2.1. Chemicals, Materials, and Solutions

Food quality synthetic dyes, tartrazine (E102), orange yellow (E110), azorubine (E122), Ponceau 4R (E124), and Brilliant Blue (E133), were from Food Colours, Piotrków Trybunalski (Poland). Their purity was assessed spectrophotometrically according to [19]. Chloroform, isopropanol, 25% aq. ammonia, ethyl acetate, pyridine, glacial acetic acid, ethanol, 1,4-dioxane, n-butanol, acetone, methanol, toluene, triethanolamine, and sodium hydroxide were from Avantor (formerly Polskie Odczynniki Chemiczne), Gliwice (Poland). The preparations analyzed in this study were purchased locally (mouthwashes) or prepared by spiking a commercial, colorless isotonic drink with appropriate dyes at concentrations 0.15, 0.45, and 0.75 mgmL^−1^. Standard solutions of dyes for TLC/densitometric analysis were prepared at the following concentrations (of pure dyes in distilled water): 0.03, 0.05, 0.15, 0.30, 0.50, 0.65, 0.80, 1.00, 1.10, 1.30, 1.45, and 1.60 mgmL^−1^. The solutions for the standard addition VIS spectrophotometric determinations of E124 and E133 dyes in mouthwashes were prepared by aqueous (blue mouthwash) or ethanolic (red mouthwash) dilution of 5.0 mL mouthwash and the appropriate volume of the dye standard solution in a 10 mL volumetric flask. The volumes of standard solutions added to the subsequent flasks were 0, 0.1, 0.2, 0.4, 0.6, 0.8, and 1.0 mL and the concentrations of standard solutions were 0.15 mgmL^−1^ (Ponceau 4R) and 0.16 mgmL^−1^ (Brilliant Blue).

### 2.2. Solid Phase Extraction (SPE)

Solid Phase Extraction was conducted with a vacuum SPE apparatus from Bioanalytic, on CRONUS SPE NH_2_ cartridges (200 mg/3 mL). SPE cartridges were conditioned with 2 × 3 mL methanol, loaded with the drink or mouthwash sample (10 mL of the product diluted with water to 50 mL), and washed with water (3 × 3 mL). The adsorbed dyes were desorbed by washing the sorbent with NaOH 0.01 molL^−1^, collected in 5 mL volumetric flasks and diluted to volume with distilled water.

### 2.3. Thin Layer Chromatography

Thin layer chromatography was performed on 10 × 20 cm standard quality silica gel 60 plates (layer thickness 0.25 mm) from Merck. Plates were developed with methanol-dichloromethane 1 : 1 (v/v) and dried at room temperature overnight prior to use. Standard solutions prepared according to Section 2.1 and the solutions of dyes isolated by SPE (Section 2.2) were spotted with the Desaga AS30 sampler equipped with a 10 *μ*L syringe (1 *μ*Lspot^−1^), 15 mm from the plate bottom edge and at 8 mm intervals, starting 10 mm from the plate edge and developed with chloroform-isopropanol-25% aq. ammonia 1 : 3 : 1 (v/v/v) as mobile phase. Plates were developed in a vertical chromatographic chamber lined with filter paper and previously saturated with the mobile phase vapor for 20 min. The development distance was 75 mm from the plate bottom edge. After development, plates were dried at room temperature (20°C), scanned, and analyzed in reflectance mode with the Desaga CD 60 densitometer at appropriate wavelengths.

### 2.4. Spectrophotometric Determination of Food Dyes in Mouthwashes by Standard Addition Method

Spectroscopic measurements were performed with the Lambda 25 UV/VIS spectrophotometer, Perkin-Elmer. Samples of mouthwashes prepared according to Section 2.1 were placed in 1 cm quartz glass cuvettes and scanned over the wavelength range 400–700 nm with the 1 nm resolution. The analytical wavelengths were 514 nm (red mouthwash) and 634 nm (blue mouthwash). Absorbances for both series of mouthwash solutions were plotted against the concentrations of the added standards and the dye concentrations without the standard addition were obtained by extrapolation of linear plots:

(1) Blue  mouthwash:  y=153.89x+0.6653R2=0.9994Red  mouthwash:  y=36.881x+0.524R2=0.9986.

## 3. Results and Discussion

### 3.1. Method Development

#### 3.1.1. Solid Phase Extraction

Liquid matrices such as beverages, drops, mouthwashes, and pharmaceutical preparations often have physicochemical properties that make their direct analysis by chromatographic or spectroscopic techniques impossible even after dilution. Their relatively high viscosity, opacity, and complex composition are the reasons why the analysis of food dyes in such samples is often a two-step process involving some isolation process prior to the actual analysis. Synthetic food dyes including those analyzed in our study have been isolated from drinks and drops by SPE on the supports such as RP-18 [4–7], cotton wool [3], polyurethane foam [10], or aminopropyl-bonded silica [5]. We have decided to use the commercial SPE columns filled with NH_2_-modified silica, recommended for normal phase extraction of polar compounds and as a weak anion exchanger (WAX) for organic anions to which the food colorants quantified in our study also belong. The p*K*_a_ of the NH_2_ functional group is around 9.8. When this sorbent is supposed to be used as an anion exchanger, the sample must be applied at a pH at least 2 units below 9.8. The pH must be at such a level that the anionic compound of interest is also charged (2 pH units above its own p*K*_a_). The elution of the anionic analyte from the sorbent may be achieved by either of three approaches: (i) neutralization of the analyte (2 pH units below its p*K*_a_); (ii) neutralization of the ionized aminopropyl group (2 pH units above its p*K*_a_); (iii) adding a different anion that competes with the analyte.

The aminopropyl-bonded silica SPE isolation of food dyes reported in [5] was based on the approach (i) with anionic food dyes neutralized with ethanolic sulfuric acid. We have, however, decided to try approach (ii) or (iii). According to our earlier research, the recovery of food colorants covered by our study from the aminopropyl-bonded silica is possible with aqueous sodium hydroxide or diluted solutions of organic basis including water soluble amines (e.g., triethanolamine, imidazole, morpholine, or 2-amine-2-methyl-1-propanol) [24]. Alternatively, we have used pH 7.4 phosphate buffered saline with added methanol. The selection of possible conditions used for the recovery of food dyes from the NH_2_ sorbent is broad and we have observed that, by careful optimization of this step, it is possible to reach a certain level of selectivity useful if the actual quantification step is based on the UV/VIS spectrophotometry. However, when the analysis involves the chromatographic separation (as it was the case in our study), selectivity of the extraction step is not crucial and the elution may be achieved easily and quickly with diluted aqueous sodium hydroxide.

#### 3.1.2. Chromatographic and Densitometric Conditions

**Figure 1:**
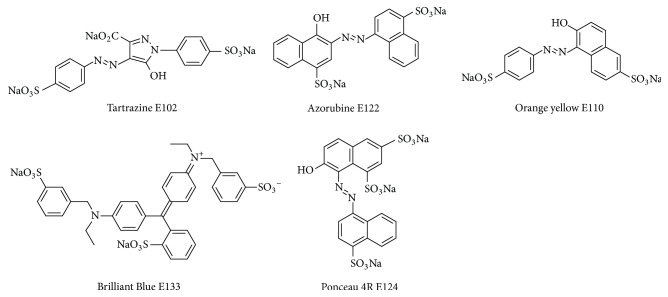
Structures of dyes E102, E110, E122, E124, and E133.

The synthetic dyes analyzed throughout this study are, from the structural point of view, sodium salts of strong (sulfonic) acids (Figure 1). On the basis of the literature data and our experience [25, 26] it was expected that such compounds, when subjected to classical RP chromatography, may exhibit low affinity to the stationary phase and tend to travel near the solvent front. It is, of course, possible to modify the RP retention of such dyes with additives such as aqueous ammonium sulfate [13, 14, 22] or suitable acidic buffers [4] but in our research we have opted for the NP chromatography or its modification known to us from our earlier research, that is, some form of Hydrophilic Interaction Liquid Chromatography- (HILIC-) type separation on the simple, cheap, and readily available unmodified silica. In order to optimize the chromatographic separation process several mobile phases have been analyzed and the *R*_*f*_ values obtained for the selected examples are presented in Table 2.

**Figure 2:**
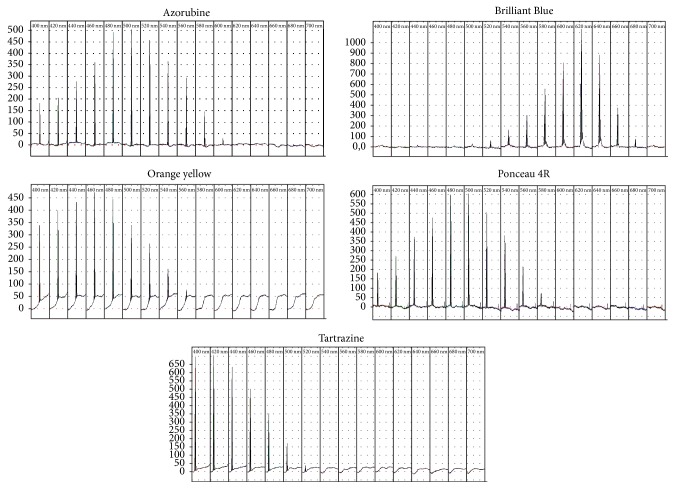
Multiwavelength scans of studied dyes.

The complete TLC separation of all dyes under investigation with a single mobile phase is not an easy task (as we knew from the literature [5, 21] and have soon found out from our own experience) and we were not entirely happy with any chromatographic system tested at this point of our study. Luckily, it is highly unlikely that as many as five dyes are used in a single preparation so it is our suggestion that a different mobile phase should be used depending on the combination of dyes present in a particular formulation. Azorubine/orange yellow and tartrazine/Ponceau 4R are two pairs of dyes that seem to be particularly difficult to separate on silica gel 60 and if any of these pairs is present we recommend using solvent system** 3** (for azorubine/orange yellow),** 8,** or** 17** (for tartrazine/Ponceau 4R). Solvent systems** 15** and** 18** can be used as the most universal mobile phases but the separation quality achieved for system** 18** is just borderline and in the case of system** 15** separation of azorubine/Brilliant Blue and orange yellow/Brilliant Blue may be incomplete. This is, however, a minor problem since the absorption ranges of dyes belonging to these pairs do not overlap (Figure 2) and their simultaneous densitometric quantification is possible in spite of the incomplete separation.

The analytical wavelengths selected on the basis of multiwavelength densitometric scanning were as follows: tartrazine 420 nm; orange yellow 460 nm; azorubine 500 nm; Ponceau 4R 500 nm; Brilliant Blue 620 nm.

### 3.2. Validation

#### 3.2.1. Calibration Plots

**Figure 3:**
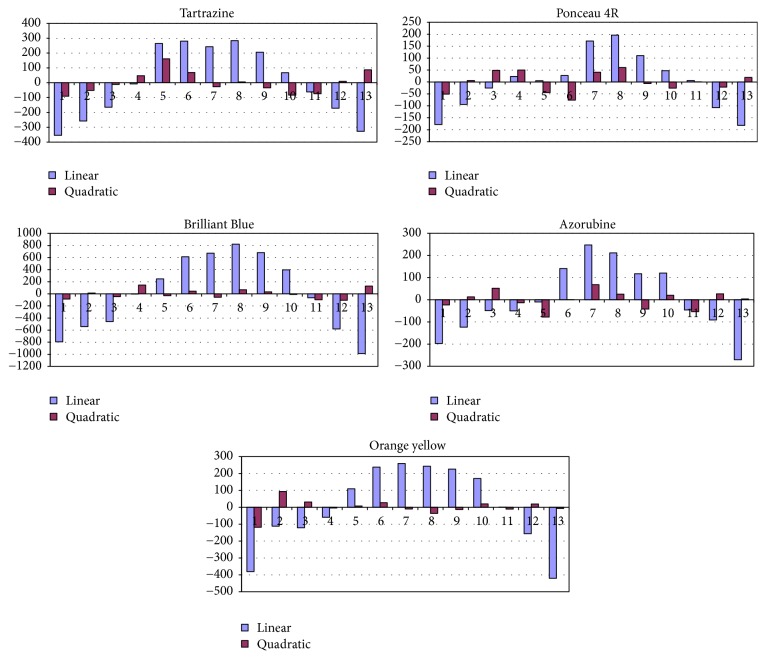
Comparison of residues for linear and quadratic calibration plots.

Standard solutions of dyes prepared according to Section 2.1 were spotted on chromatographic plates and developed according to Section 2.3. Chromatograms were developed and scanned according to Section 2.3 and the surfaces of chromatographic peaks were plotted against the amount of dyes. The linear and quadratic calibrating plots were generated and compared by means of *R* values and nonnumerical analysis of residues according to [27] (Figure 3). It was concluded that the quadratic calibration lines give a better fit and the resulting equations are presented in Table 3.

#### 3.2.2. Limit of Quantification (LOQ)

The quantification limits of our method is difficult to determine. The limits for the TLC/densitometry step, determined according to [27] (Section  4.8.a) are given in Table 3 but the SPE sample pretreatment step makes it possible to concentrate the analytes, as we have estimated earlier, by two orders of magnitude, depending on the size of the SPE column and the composition of the eluent, with the experiment timing as the main limitation (sample flow rate through the sorbent must not be too high to ensure the complete adsorption of the analyte). Taking into consideration the size of SPE columns that we have used and the extraction time we have estimated the overall LOQ at* ca.* 1 mgL^−1^ or less.

#### 3.2.3. Specificity

Reflectance UV/VIS spectra of the dyes isolated from analyzed samples and processed chromatographically were obtained directly from the TLC spots (three different places within each spot) and compared to those of the standards to prove the identity and purity of dyes separated by SPE and thin layer chromatography.

#### 3.2.4. Precision and Accuracy

Precision of the proposed method was tested on two levels: repeatability and intermediate precision ([27], Sections  4.5.1 and  4.5.2). The method accuracy was tested according to [27] (Section  4.6), using spiked drink samples (Table 4) and real mouthwash samples, simultaneously analyzed by UV/VIS spectrophotometry according to the procedure, described in Section 2.4 (Table 5).

A colorless drink, containing water, sugar, citric acid, magnesium, sodium citrate, natural lemon flavor, potassium sorbate, dimethyl dicarbonate, ester gum, and vitamins B1, B6, B12, and E, was spiked with dyes at 3 levels of concentrations (0.15, 0.45, and 0.75 mgmL^−1^). 10 mL samples of the drink were analyzed according to Sections 2.2 and 2.3. Dye recoveries and coefficients of variation (CV) are given in Table 4.

Two brands of analyzed, commercial mouthwashes (red and blue) had the following compositions:

1. Red mouthwash: glycerin, ethanol, water, benzyl alcohol, chlorhexidine, chlorobutanol, citral, citronellol, sodium docusate, eugenol, limonene, linalool, menthol, and E124.
2. Blue mouthwash: water, ethanol, sodium benzoate, cocamidopropyl betaine, sodium saccharine, zinc chloride, propylene glycol, sodium fluoride, olaflur, sodium chloride, glycerin, and E133.

The results of spectrophotometric and chromatographic analyses of commercial mouthwashes are given in Table 5.

## 4. Conclusion

The proposed method of isolation and quantification of food dyes is easy, accurate, and precise enough for the routine analysis of the most widely used colorants in liquid matrices. Other components of liquid preparations, including surfactants, vitamins, preservatives, and flavors, do not interfere. Due to the possibility of using different SPE and TLC eluents tested in our study, the proposed method has a high potential for further developments, including colorants other than the aforementioned ones, analysis of complex dye mixtures, and matrices of different composition.

## Figures and Tables

**Table 1:** Selected references on TLC separation and/or quantification of synthetic food dyes (including some illegal or delisted examples).

Dyes	Sorbent	Eluent	Detection method	Ref.
Amaranth, indigotine, tartrazine, Ponceau 4R, orange yellow, Allura Red, Brilliant Blue, food green 3	Silica gel RP18	Methanol-acetonitrile-5% aq. Na_2_SO_4_ (3 : 3 : 10, v/v/v) Methanol-butanone-5% aq. Na_2_SO_4_ (1 : 1 : 1, v/v/v)	Densitometry	[14]

Detection of illegal dyes	RP-18	Methanol-acetonitrile-5% aq. Na_2_SO_4_ (3 : 3 : 10, v/v/v) Methanol-butanone-5% aq. Na_2_SO_4_ (1 : 1 : 1, v/v/v) Methanol-butanone-5% aq. Na_2_SO_4_ (1 : 1 : 1, v/v/v)	Mass spectroscopy	[13]

Tartrazine, azorubine, orange yellow	NH_2_	*i*-PrOH-Et_2_O-NH_3_ (2 : 1 : 1 v/v/v)	Video-scanning	[15]

Ponceau 4R, tartrazine, orange yellow	Silica gel 60	*i*-PrOH-NH_3_-H_2_O (10 : 1 : 1 v/v/v)	Sorbent scraping, extraction, and spectrophotometry	[11]

Indigotine, Ponceau 4R, orange yellow, tartrazine, amaranth	MgO	Sodium citrate 15% aq.-methanol (4 : 1 v/v)	Visual	[20]

Amaranth, tartrazine, orange yellow	Silica gel	*n*-butanol-ethanol-H_2_O-NH_3_(10 : 5 : 5 : 2 v/v/v/v)	Visual	[3]

Tartrazine, Allura Red	RP-18W	Methanol-citric buffer pH 3.5 (45 : 55 v/v)	Densitometry	[4]
Ponceau 4R	RP-18W	Tetrahydrofuran-acetic buffer pH 3.5 (45 : 55 v/v)		
Brilliant Blue	CN	1,4-Dioxane-acetic buffer pH 3.5-H_2_O (25 : 20 : 55 v/v/v) + 0.025 M sodium octane-1-sulfonate		
Azorubine	RP-18W	Acetone-acetic buffer pH 3.5 (4 : 6 v/v)		

Patent blue	CN	Tetrahydrofuran-acetic buffer pH 3.5-H_2_O (25 : 20 : 55 v/v/v) + 0.025 M diethylamine	Densitometry	[4]
Quinoline yellow	RP-18W	1,4-Dioxane-acetic buffer pH 3.5 (1 : 9 v/v)		

Amaranth, tartrazine, Brilliant Blue	Scolecite	Acetone	Visual	[21]
	Silica gel G			

Tartrazine, orange yellow, quinolone yellow, amaranth, Ponceau 6R, erythrosine, indigotine, Brilliant Blue, brilliant black	RP-18	Water-ethanol-(NH_4_)_2_SO_4_ aq. Water-acetone-(NH_4_)_2_SO_4_ aq.	Visual	[22]

Fat soluble dyes	Al_2_O_3_	Petroleum ether-CCl_4_ (1 : 1 v/v)	Visual	[8]
	Starch impregnated with paraffin or vegetable oil	MeOH-H_2_O-AcOH (80 : 15 : 5 v/v/v)		
Water soluble dyes	Polyamide powder	NH_3_-methanol-H_2_O 5 : 15 : 80 (v/v/v)		

Fat soluble dyes	Silica gel G	Benzene	Visual	[23]
Water soluble dyes	Silica gel G	*i*-BuOH-HCOOH-H_2_O (25 : 10 : 10 v/v/v)		
		*i*-BuOH-AcOH-H_2_O (25 : 10 : 10 v/v/v)		
		Butanone-methyl isobutyl ketone-AcOH-H_2_O (20 : 10 : 10 : 5 v/v/v/v)		
		*i*-BuOH-*i*-PrOH-NH_3_ aq. (1 : 1 : 1 v/v/v)		
		Butanone-pyridine-H_2_O (4 : 1 : 1 v/v/v)		

Naphthol yellow, tartrazine, Ponceau 4R, amaranth, erythrosine, rhodamine B, indigotine, patent blue	Silica gel G	EtOH-*n*-BuOH-H_2_O (9 : 2 : 1 v/v/v)	Sorbent scraping, extraction, and spectrophotometry	[12]
			Densitometry	
			Spectral reflectance	

Tartrazine, orange yellow, amaranth, Ponceau SX, erythrosine, Allura Red, Brilliant Blue, food green 3	Silica gel 60	*n*-BuOH-AcOH-H_2_O (4 : 1 : 5 v/v/v)	Densitometry	[5]
		*n*-BuOH-EtOH-H_2_O-NH_3_ (10 : 5 : 5 : 2 v/v/v/v)		

**Table 2:** *R*_*f*_ values of food dyes, selected mobile phases.

	*R*_*f*_^1^	*R*_*f*_^2^	*R*_*f*_^3^	*R*_*f*_^4^	*R*_*f*_^5^	*R*_*f*_^6^	*R*_*f*_^7^	*R*_*f*_^8^	*R*_*f*_^9^	*R*_*f*_^10^	*R*_*f*_^11^	*R*_*f*_^12^	*R*_*f*_^13^	*R*_*f*_^14^	*R*_*f*_^15^	*R*_*f*_^16^	*R*_*f*_^17^	*R*_*f*_^18^	*R*_*f*_^19^	*R*_*f*_^20^
Azorubine	0.50	0.62	0.28	0.77	0.43	0.34	0.42	0.47	0.58	0.74	>0.95	0.91	>0.95	0.75	0.70	0.92	0.63	0.49	0.88	0.88
Tartrazine	0.21	0.36	0	0.61	0.08	0.11	0.04	0.12	0.25	0.50	>0.95	0.89	>0.95	0.44	0.42	0.45	0.28	0.06	0.70	0.78
Ponceau 4R	0.20	0.40	0	0.63	0.06	0.15	0.07	0.20	0.30	0.56	>0.95	0.89	>0.95	0.48	0.59	0.49	0.21	0.13	0.73	0.75
Brilliant Blue	0.37	0.43	0	0.64	0.19	0.16	0.17	0.33	0.37	0.56	>0.95	0.89	>0.95	0.43	0.74	0.56	0.30	0.19	0.64	0.73
Orange yellow	0.48	0.60	0.11	0.75	0.42	0.29	0.35	0.44	0.54	0.69	>0.95	0.91	>0.95	0.76	0.80	0.82	0.59	0.42	0.85	0.91

^1^CHCl_3_-*i*-PrOH-NH_3_ 25% aq. (1 : 3 : 1 v/v/v); ^2^EtOAc-pyridine-H_2_O (11 : 5 : 4 v/v/v); ^3^CHCl_3_-*i*-PrOH-AcOH (2 : 6 : 1 v/v/v); ^4^EtOAc-pyridine-H_2_O (2 : 1 : 1 v/v/v); ^5^CHCl_3_-*i*-PrOH-NH_3_ 25% aq. (2 : 6 : 1 v/v/v); ^6^EtOAc-pyridine-H_2_O (12 : 5 : 3 v/v/v); ^7^CHCl_3_-*i*-PrOH-H_2_O (2 : 6 : 1 v/v/v); ^8^CHCl_3_-*i*-PrOH-H_2_O (1 : 3 : 1 v/v/v); ^9^(a) CHCl_3_-*i*-PrOH-H_2_O (2 : 6 : 1 v/v/v); (b) EtOAc-pyridine-H_2_O (11 : 5 : 4 v/v/v); ^10^EtOAc-EtOH-H_2_O (75 : 35 : 30 v/v/v); ^11^1,4-dioxane-H_2_O (2 : 1 v/v); ^12^EtOH-n-butanol-H_2_O (9 : 1 : 2 v/v/v); ^13^Acetone-MeOH-NH_3_ 25% aq. (8 : 4 : 1 v/v/v); ^14^EtOAc-MeOH-H_2_O-AcOH (65 : 33 : 11 : 1); ^15^MeOH-toluene (11 : 9 v/v); ^16^MeOH-toluene-AcOH (55 : 40 : 5 v/v/v); ^17^EtOAc-EtOH-NH_3_ 25% aq. (75 : 45 : 15 v/v/v); ^18^EtOAc-*i*-PrOH-H_2_O (75 : 35 : 30 v/v/v); ^19^EtOAc-MeOH-H_2_O-triethanolamine (65 : 33 : 11 : 1 v/v/v/v); ^20^*i*-PrOH-NH_3_ 25% aq. (8 : 3 v/v).

**Table 3:** Regression coefficients (*y* = *ax*^2^ + *bx* + *c*) with confidence intervals. Analytical wavelengths selected on the basis of densitometric scanning (Figure 2).

	*a* ± *t* (95%, 10) *s*_*a*_	*b* ± *t* (95%, 10) *s*_*b*_	*c* ± *t* (95%, 10) *s*_*c*_	*R* ^2^	LOQ
					*µ*gspot^−1^
Orange yellow	−834.01 ± 122.52	2997.32 ± 205.55	120.61 ± 64.91	0.9978	0.09
Azorubine	−595.56 ± 115.42	3498.70 ± 186.89	24.76 ± 56.96	0.9992	0.08
Brilliant Blue	−2603.68 ± 242.76	9228.31 ± 378.86	88.79 ± 111.29	0.9993	0.06
Ponceau 4R	−504.84 ± 137.75	2619.17 ± 206.92	52.42 ± 58.50	0.9982	0.07
Tartrazine	−836.85 ± 191.30	3620.63 ± 320.95	92.62 ± 101.35	0.9971	0.04

**Table 4:** Dye contents in spiked preparations determined by SPE/TLC/densitometry.

	Dye	Expected dye content	Recovered dye content	CV [%]	Recovery [%]
		*µ*g*µ*L^−1^	*µ*g*µ*L^−1^	*n* = 3	
Blue drink	E133	0.15	0.14	3.1	93.3
		0.45	0.41	2.7	91.1
		0.75	0.73	3.3	97.3

Red drink	E122	0.15	0.14	1.6	93.3
		0.45	0.42	2.5	93.3
		0.75	0.71	3.0	94.7

Red drink	E124	0.15	0.13	4.1	86.7
		0.45	0.43	2.9	95.6
		0.75	0.72	2.3	96.0

Yellow drink	E110	0.15	0.14	2.8	93.3
		0.45	0.42	3.2	93.3
		0.75	0.74	2.7	98.7

Yellow drink	E102	0.15	0.13	3.3	86.7
		0.45	0.42	2.9	93.3
		0.75	0.73	3.4	97.3

**Table 5:** Dye concentrations in real samples determined by UV/VIS spectrophotometry or SPE followed by TLC/densitometry.

	Dye	UV/VIS	TLC/densitometry			TLC/densitometry		
			Day 1, Analyst 1			Day 2, Analyst 2		
		Dye content *µ*gmL^−1^	Dye content *µ*gmL^−1^	CV [%]	Recovery [%]	Dye content *µ*gmL^−1^	CV [%]	Recovery [%]
Blue mouthwash	E133	8.6	7.7	2.23	89.5	7.8	3.62	90.7
Red mouthwash	E124	28.0	26.0	1.02	92.3	25.6	1.89	91.4
